# Mapping the Impact of Non-Tectonic Forcing mechanisms on GNSS measured Coseismic Ionospheric Perturbations

**DOI:** 10.1038/s41598-019-54354-0

**Published:** 2019-12-09

**Authors:** Mala S. Bagiya, A. S. Sunil, Lucie Rolland, Srinivas Nayak, M. Ponraj, Dhanya Thomas, Durbha Sai Ramesh

**Affiliations:** 1 Indian Institute of Geomagnetism (DST), Navi Mumbai, India; 2 Université Côte d’Azur, OCA, CNRS, IRD, Géoazur, Sophia-Antipolis, Valbonne, France

**Keywords:** Natural hazards, Solid Earth sciences, Space physics

## Abstract

Global Navigation Satellite System (GNSS) measured Total Electron Content (TEC) is now widely used to study the near and far-field coseismic ionospheric perturbations (CIP). The generation of near field (~500–600 km surrounding an epicenter) CIP is mainly attributed to the coseismic crustal deformation. The azimuthal distribution of near field CIP may contain information on the seismic/tectonic source characteristics of rupture propagation direction and thrust orientations. However, numerous studies cautioned that before deriving the listed source characteristics based on coseismic TEC signatures, the contribution of non-tectonic forcing mechanisms needs to be examined. These mechanisms which are operative at ionospheric altitudes are classified as the i) orientation between the geomagnetic field and tectonically induced atmospheric wave perturbations ii) orientation between the GNSS satellite line of sight (LOS) geometry and coseismic atmospheric wave perturbations and iii) ambient electron density gradients. So far, the combined effects of these mechanisms have not been quantified. We propose a 3D geometrical model, based on acoustic ray tracing in space and time to estimate the combined effects of non-tectonic forcing mechanisms on the manifestations of GNSS measured near field CIP. Further, this model is tested on earthquakes occurring at different latitudes with a view to quickly quantify the collective effects of these mechanisms. We presume that this simple and direct 3D model would induce and enhance a proper perception among the researchers about the tectonic source characteristics derived based on the corresponding ionospheric manifestations.

## Introduction

Imprints of seismic forcing on the ionosphere during earthquake events are extensively studied using Global Positioning System (GPS) – Total Electron Content (TEC) measurement technique^1–7^. GPS is part of the Global Navigation Satellite System (GNSS), being operated by the US. The GPS recorded coseismic ionospheric perturbations (CIP), are considered to be very useful in providing the spatio-temporal manifestations of tectonic forcing in the ionosphere. Tracing back to the source of CIP, the CIP evolution surrounding the epicenter highly depends on the source characteristics in terms of crustal deformation pattern which evolves mainly during the rupture propagation^5,8^.

The ground based measurement of crustal deformation has a stringent requirement of availability of the sounding probe at or very near to the deformed area e.g. GPS receiver in precise positioning mode. With the advent of space based technology like InSAR, it is easy to acquire this information, provided successive satellite passes coincide over the earthquake occurrence region^9^. But for offshore earthquakes, InSAR also fails in providing reliable measurements of crustal deformation. In other cases, one may have to rely on the model based estimation constrained by the available ground measurements, for e.g. 11 March 2011 Tohoku earthquake^10^.

Therefore, an alternative tool needs to be explored that can provide reliable information about the tectonic source characteristics. In recent times, efforts are on to identify the tectonic source characteristics, such as rupture propagation direction, crustal deformation pattern and thrust orientations, based on the azimuthal distribution of near field CIP as recorded by GPS^2,5–7,11,12^. So, is it always possible to characterize the tectonic source using GNSS measured near field CIP? It seems possible, but there lays the difficulty in terms of non-tectonic forcing mechanisms that act upon the CIP evolution at ionospheric altitudes^5–7,12^.

The basic physical mechanism responsible for the generation of CIP is the redistribution of ionospheric plasma by tectonically induced neutral atmospheric wave perturbations. These perturbations are mainly induced by the coseismic crustal deformation. The major effective non-tectonic forcing mechanisms at ionospheric altitudes are the (i) orientation between the ambient geomagnetic field and seismic induced neutral wave perturbations^12–15^ (ii) orientation between the moving satellite line of sights and the wave perturbations^6,7,16^ and (iii) ambient ionospheric electron density gradient. The effects of the geomagnetic field-neutral wave orientation on near field CIP evolution and subsequent North-South asymmetry has been estimated with a realistic simulation using a case study based on the Van earthquake^12^. Bagiya *et al*.^6,7^ further investigated the CIP efficiency in identifying the tectonic source characteristics, during the 12 May 2015 Nepal and 13 November 2016 Kaikoura earthquakes, by introducing an elementary satellite geometry factor which estimates the wave phase cancellation effects on CIP amplitudes arising from the varying satellite geometry. Based on these studies, it could be stated that GNSS measured near field CIP azimuthal distribution offers a reasonable alternative opportunity to determine the seismic source characteristics from the ionosphere, provided non-tectonic forcing mechanisms favor the evolution of CIP.

Despite these individual case studies, no attempt has been made so far to map the combined effects of the non-tectonic forcing mechanisms on GNSS measured CIP. We propose, for the first time, a simple 3D geometrical model to estimate the collective non-tectonic effects on the evolution of CIP within ~600 km of radial distance from the ionospheric projection of seismic source. The constraint of ~600 km radial distance is explained later in the text. Firstly, we define non-tectonic forcing mechanism involving the geomagnetic field variations as Geomagnetic field - Neutral wave orientation Factor (GNF) and the one which involves the satellite geometry effects as Satellite Geometry Factor (SGF)^6^. The effects of ambient electron density variations are estimated as Electron Density Factor (EDF).

We first estimate the GNF and SGF individually, based on the propagation of seismo-acoustic wave perturbations in 3D atmospheric space and time^14,17^, for a Trial earthquake source near the Main Himalayan Thrust (MHT). Since satellite geometry effects significantly depend on the location of the ground receiver, the SGF is estimated for various GPS stations assumed within and ~600 km surrounding the Trial tectonic source. The EDF is estimated based on the TEC variations provided by the International GNSS Service (IGS) TEC maps. We then multiply the GNF, SGF, and EDF and introduce a factor that estimates the collective non-tectonic effects in 3D space for the Trial tectonic source. This factor is termed as the Non-Tectonic Forcing Mechanisms (NTFM) factor and the 3D computation model is captioned as the NTFM model.

We further assess the performance of the proposed NTFM model during actual earthquake events. We consider here five events: (i) 01 April 2014 Iquique earthquake (ii) 16 September 2015 Illapel earthquake (iii) 25 April 2015 Nepal earthquake (iv) 11 April 2012 Sumatra earthquake (Mw 8.6) and (v) 13 November 2016 New Zealand earthquake. The selection of these earthquakes is made very thoughtfully. No studies so far reported the latitudinal variations of non-tectonic forcing mechanisms. To generalize the proposed model application for earthquakes occurring at various geographical locations, we validate the NTFM model using these selected earthquake events that occurred at different latitudes. In a brief, the present approach is first of its kind in proposing a 3D model that can estimate the combined effects of non-tectonic forcing mechanisms on the evolution of GNSS measured CIP at various latitudes. We believe that this simple model would assist the researchers with a reasonably clear notion about the tectonic source characteristics derived based on the corresponding ionospheric perturbations.

## Methodology

### Modeling of seismo-acoustic rays in 3D space and time

The upper atmosphere responds profoundly to the tectonic energy within 1 to 10 mHz. This frequency domain contains both acoustic as well as gravity waves. The acoustic wave propagation is significantly affected by the temperature of a medium. This could be understood from the Eq. (1)^18^ which estimates the acoustic wave velocity. Accordingly, the seismo-acoustic wave propagation in the overlying atmosphere depends highly on the ambient atmospheric temperature.

1 $${\rm{V}}=\sqrt{\frac{\gamma RT}{M}}$$

Here γ is the ratio of specific heat capacities, R is the universal gas constant, T is the temperature and M is the molecular mass density. The varying temperature and density cause the refraction of seismo-acoustic waves with atmospheric altitudes by changing their velocity. We model the propagation of seismo-acoustic rays (k) in space and time based on the wave refraction phenomenon. For this, the neutral atmospheric temperature and density are obtained from the NRLMSISE-00^19^ model. The acoustic rays are modeled up to the peak ionospheric electron density altitude obtained from the IRI-2016 empirical model^20^.

### 3D model to quantify the effects of non-tectonic forcing mechanisms on the manifestations of GNSS measured CIP

We, firstly, model the Geomagnetic field - Neutral wave orientation Factor (GNF). This factor is similar to the ionospheric coupling factor (α) by Calais *et al*.^13^. In order to do so, we extract the values of magnetic inclination and declination corresponding to the geographic location of a tectonic source and nearby region based on the IGRF-12 model^21^. Using these values, we estimate the geomagnetic field unit vector and denote it as ***b***. The unit vector ***b*** and the seismo-acoustic rays, modeled using the method described in (1), are the main ingredients for estimating the GNF. The equation^15,16^ to calculate the GNF as follows.

2 $$GNF(\lambda ,\varphi ,h)={\boldsymbol{k}}(\lambda ,\varphi ,h)\cdot {\boldsymbol{b}}(\lambda ,\varphi ,h)$$

Where λ, φ, and h depict the geographic longitude, geographic latitude and terrestrial altitude respectively. From Eq. (2), the GNF can vary from −1 to 1 (cosine function).

In the second step, we estimate the non-tectonic forcing effects arising due to the ambient electron density variations. For this, we use the International GNSS Service (IGS) TEC maps which provide a snapshot of the global ionosphere at every 15 min, 1 hr, and 2 hrs^22,23^. These maps are prepared by the IGS iono working group using the global IGS TEC observations (http://cdaweb.gsfc.nasa.gov). In maps, TEC is estimated as vertical TEC at ionospheric piercing point (IPP) altitude of 400 km and at latitude × longitude grid of 2.5° × 5.0° within the spatial domain of −87.5° to 87.5° latitudes and −180° to 180° longitudes. The satellite elevation cutoff is 10°. We extract the TEC values for our preferred geographical grid at 15 min interval and interpolate it to obtain the variations at finer grid points. The interpolated TEC values are then normalized and denoted as, Electron Density Factor, EDF(λ, φ, h_IPP_). Since the obtained TEC variations are at fixed IPP altitude of 400 km, the h is presented as h_IPP_.

In the third step, the effects of moving satellite geometry are computed. The orientation between the satellite line of sight (LOS) and vertically propagating seismo-acoustic rays has significant effects in terms of phase integration of the propagating waves. Inspired by Georges and Hooke, (1970)^6^, Bagiya *et al*.^16^ invoked an elementary version of satellite geometry factor (SGF) to account for the wave phase cancelation effects during varying GPS satellite geometry. This factor was formulated as,

3 $$SGF(\lambda ,\varphi ,h)=\frac{{\boldsymbol{k}}({\rm{\lambda }},{\rm{\varphi }},{\rm{h}})\cdot {\boldsymbol{r}}({\rm{\lambda }},{\rm{\varphi }},{\rm{h}})}{cos\chi }$$

where r is the satellite LOS wave vector and χ is the satellite zenith angle. We modify the algebraic expression for the SGF and reformulate it as follows:

4 $$SGF(\lambda ,\varphi ,h)=exp(\,-\,\frac{{\boldsymbol{k}}({\rm{\lambda }},{\rm{\varphi }},{\rm{h}})\cdot {\boldsymbol{r}}({\rm{\lambda }},{\rm{\varphi }},{\rm{h}})}{cos\chi })$$

We consider the absolute values of ***k***(λ, φ, h) •***r***(λ, φ, h)in Eq. (4) and thus the SGF can vary from 0 to 1. The requirement for the algebraic transformation of Eq. (3) is discussed further in the text. We estimate the SGF for near field GNSS stations for all possible satellite LOS in view. Finally, we integrate the effects of GNF, SGF, and EDF by using the algebraic multiplication and propose a simplified NTFM factor as follows.

5 $$NTFM(\lambda ,\varphi ,h)=GNF(\lambda ,\varphi ,h)\times EDF(\lambda ,\varphi ,{h}_{IPP})\times SGF(\lambda ,\varphi ,h)$$

Here × denotes the algebraic multiplication. The computations described under Eqs. (1, 2, 4, and 5) are essentially named as the 3D NTFM model. Since electron density measurements from each ionospheric altitudes over a specific geographical location are not always available, we use the IGS map derived TEC for computing the NTFM factor at various ionospheric altitudes.

### Coseismic crustal displacement fields based on the static solution

The coseismic crustal deformation in terms of horizontal and vertical displacement fields are computed based on daily position estimates of the permanent GPS sites. The GAMIT/GLOBK software package is used to estimate the daily positions of GPS sites^24,25^. In the GAMIT processing, a loosely constrained daily solution is computed which further processed with GLOBK to obtain an accurate solution. The analysis has been performed using GPS measured positioning time series over 41 days (20 days prior to the event, event day and 20 days after the event) in each case. The difference in position coordinates on the day before and day after the earthquake provides the respective coseismic displacement fields.

### Coseismic Ionospheric Perturbations (CIP) from GPS-TEC observations

GPS data in RINEX format from permanent GPS sites have been analyzed based on the following formula to estimate the slant Total Electron Content (sTEC),

6 $$sTEC=\frac{1}{40.3}(\frac{{f}_{1}^{2}{f}_{2}^{2}}{{f}_{1}^{2}-{f}_{2}^{2}})({L}_{1}{\lambda }_{1}-{L}_{2}{\lambda }_{2})$$

where *f*_1_and *f*_2_ are the carrier frequencies (1575.42 MHz and 1227.60 MHz respectively), *L*_1_and *L*_2_ are the carrier phases and *λ*_1_ and *λ*_2_ are the corresponding wavelengths.

The data sampling interval is 30 s. An elevation mask of 30° is applied to exclude the low elevation satellite measurements. The sTEC is further converted to vertical TEC using the mapping function M as follows^26^,

7 $$vertical\,TEC=M\times sTEC$$

$$M={[1-{(\frac{\cos (E)}{1+h/{R}_{E}})}^{2}]}^{1/2}$$

where *R*_*E*_ denotes the Earth’s mean radius, *E* is the satellite elevation angle, and *h* is the IPP height. To highlight the CIP, the obtained vertical TEC is applied with a bandpass filter of 1 to 10 mHz which removes long period trends in vertical TEC.

### Implantation of 3D NTFM model for Trial earthquake source

**Figure 1:**
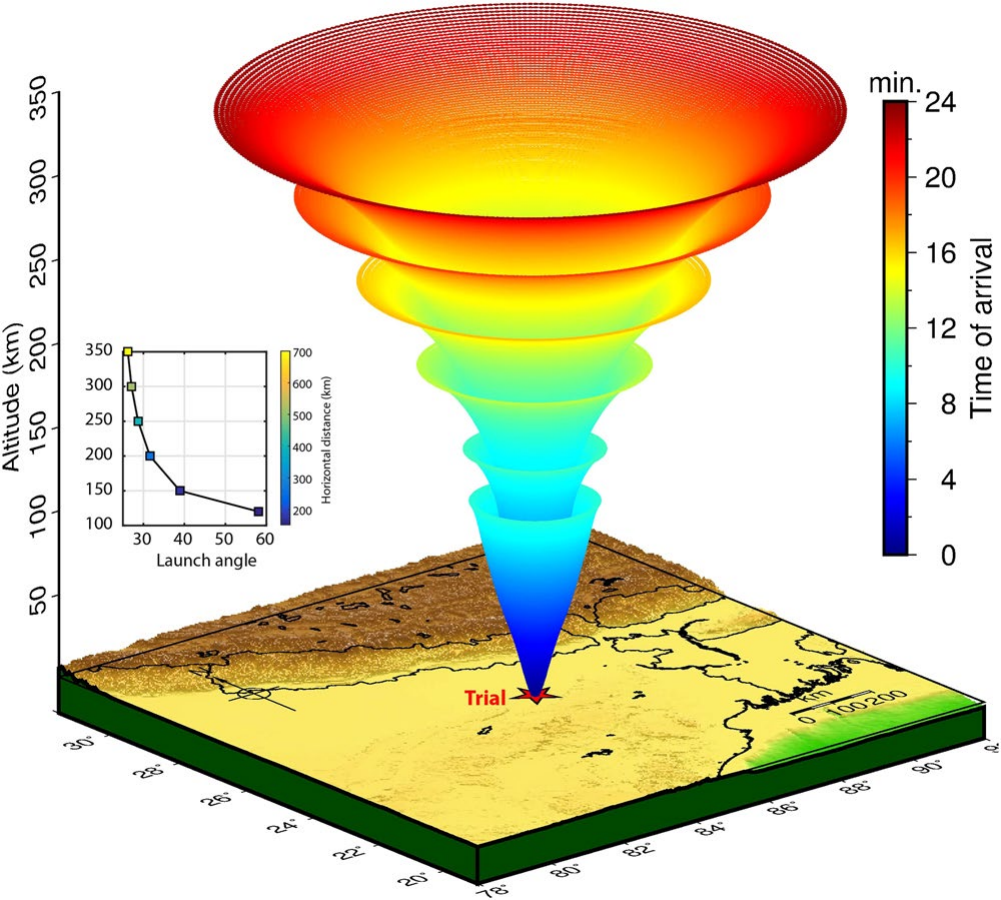
Propagation of seismo-acoustic waves in 3D space with time from the Trial seismic source assumed at 25°N 85°E. The propagation of six rays modeled at six different launch angles is shown. The first ray is launched at an angle of ~58° that is the threshold angle at 120 km altitude. The rays with launch angles higher than this refract downward while those of lower than this propagate further upward. Similarly, the second ray is launched at an angle of ~38.8° that is the threshold propagation angle at 150 km altitude and so on. The inset shows the variation of the threshold angle and maximum horizontal distance along with the atmospheric altitudes. The figure is prepared using the Generic Mapping Tools (GMT) 5.4.4^43^.

We consider a Trial earthquake source at the northern low latitude (Fig. 1) and compute the effects of non-tectonic forcing mechanisms at various ionospheric altitudes over the epicenter through our proposed 3D model. The Trial earthquake is assumed on 02 March 2010 at 12:00 UT. It should be noted that this day was a geomagnetically quiet day. To start with, we estimate the altitudinal variations of acoustic wave velocity in the atmosphere over the Trial source location and present in Fig. S1 along with the neutral atmospheric temperature profile obtained from the NRLMSISE-00 model. Using the estimated acoustic wave velocity profile, we model the propagation of seismo-acoustic rays in space with time based on the method described in (1). Figure 1 demonstrates the modelled propagation of six different rays launched at zenith angles (angle from the vertical) of ~58°, ~38.8°, ~31.6°, ~28.7°, ~27.1°, and ~26.2° which are the respective threshold (i.e. beyond which rays refract downward) angles for ray propagation at ionospheric altitudes of 120 km, 150 km, 200 km, 250 km, 300 km, and 350 km. The inset shows the variation of the threshold angle and maximum horizontal distance as a function of the atmospheric altitudes. It should be noted that the NTFM model computation is performed using the rays modeled at every 0.1° resolution within the estimated threshold angle at a given specific altitude. The 2D manifestation of rays modeled at every 0.1° for all possible launch angles (till ~58° from the zenith) could be seen in Fig. S3 and S4. Further, at an altitude of 350 km, the seismo-acoustic rays can propagate to the radial distance of ~680 km from the ionospheric projection of the Trial earthquake source thus we restrict the proposed model computation for a distance of ~600 km surrounding the tectonic source.

We now compute the GNF, EDF, and SGF. The GNF and SGF are computed based on the obtained ray traces in 3D space and time. The GNF depends on two parameters (Eq. 2), the geomagnetic field and atmospheric temperature through acoustic wave velocity. The acoustic wave velocity, as mentioned, is a function of ambient temperature that varies with time of the day, season, solar activity, and latitude as well. On the contrary, the geomagnetic field exhibits secular variations but it varies significantly with latitudes. This could be verified from Fig. S2 which shows the latitudinal variations of magnetic inclination (dip) over the globe obtained from the IGRF-12 model. The location of the Trial epicenter is also shown in the figure. According to the variations of magnetic inclination, we classify the region between 0º–~ 40° as low, ~ 40°–~ 60° as mid and ~60° and above as high latitude region^27–29^.

**Figure 2:**
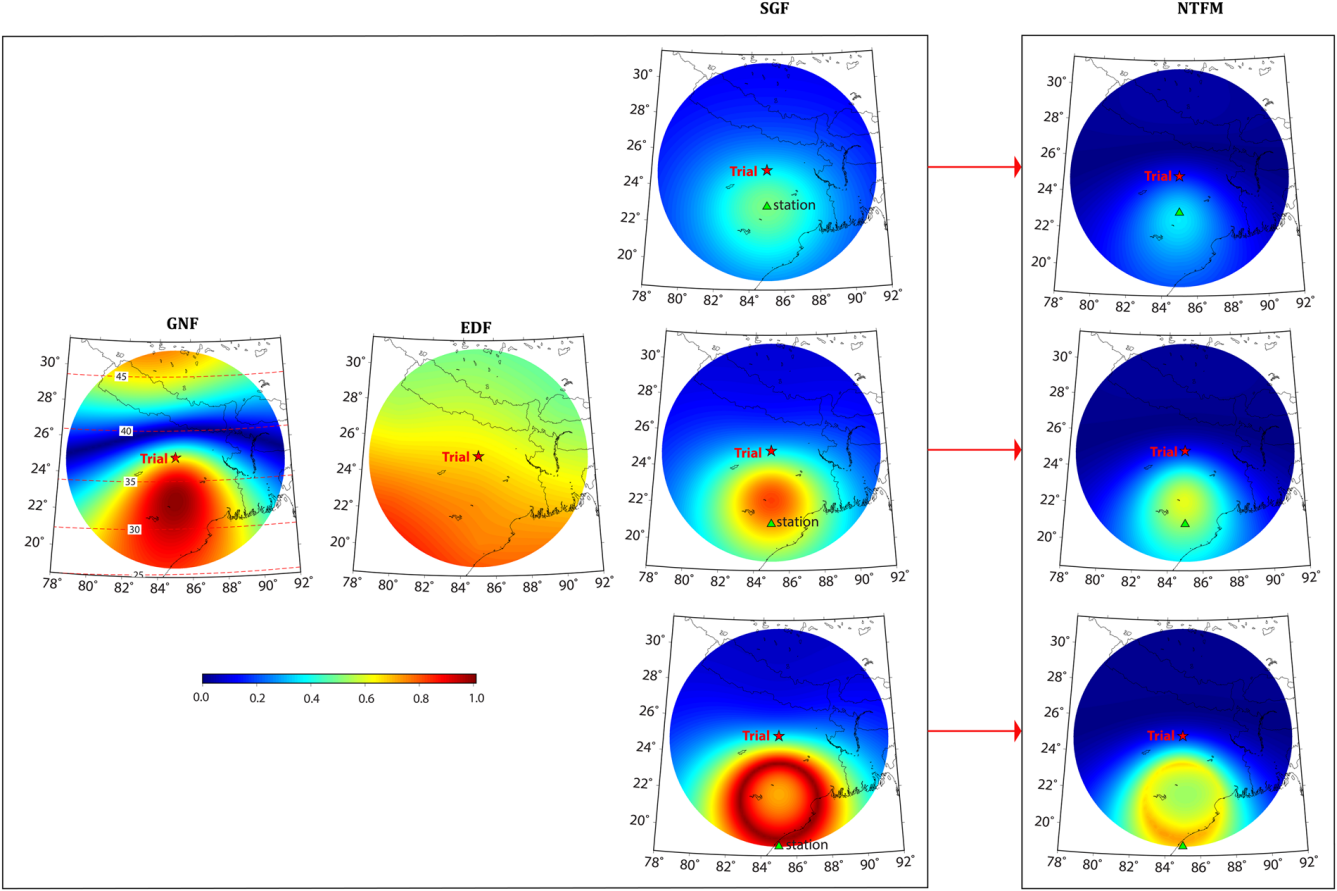
2D manifestation of GNF, EDF, SGF and their collective effects as NTFM factor at ionospheric altitude of 350 km for the Trial seismic source. The figure is prepared using the GMT 5.4.4^43^.

Figure 2 shows the GNF estimated at ionospheric peak density altitude of 350 km over the Trial seismic source. The red star in the figure demonstrates the ionospheric projection of the Trial epicenter. The 2D schematic in Fig. S3 represents the interaction between the seismo-acoustic rays and geomagnetic field for the Trial seismic source. Since the source is considered in the northern hemisphere, the geomagnetic field vectors are inclined down from the horizontal as shown in the figure. The angle between ***k*** and ***b*** is ~180° towards the equator (case-I) and thus the absolute values of GNF ≅ 1 (Eq. 2). Near to the Trial source, the angle is more than 90° (case-II) while both the vectors become almost perpendicular further poleward (case-III). The GNF approaches 0 value for perpendicular geometry. We present the absolute values of GNF in Fig. 2. It has to be noted that GNF varies from 0 to 1 which shows respectively unfavorable to favorable conditions for the manifestations of ionospheric perturbations from the geomagnetic field geometry point of view. Therefore, GNF favors the equatorward evolution and propagation of CIP in the Trial case.

The latitudinal gradient of ambient ionospheric density plays an important role in manifesting the amplitude of ionospheric perturbations. In the case of higher electron density, the perturbations can evolve with higher amplitudes. However, the reduced density results in perturbations with smaller amplitudes (e.g. during nighttime). In order to estimate the EDF, we extract TEC from the IGS map with a latitude-longitude grid of 18°N–31°N and 78°E–92°E on 02 March 2010 at 12:00 UT and subsequently interpolate it to obtain the TEC variations at a finer resolution. The EDF is estimated by normalizing the TEC and therefore the variations are positive and less than 1 (Fig. 2). The figure demonstrates that the electron density gradient is relatively higher south of the Trial epicenter and thus the CIP in the south may evolve with higher amplitudes.

It should be noted that TEC in IGS maps is computed at an ionospheric altitude of 400 km. In view of the unavailability of electron density measurements at specific ionospheric altitudes, the obtained TEC variations are considered to represent the spatial variation of ionization density at other altitudes as well. It is understood that though the ionospheric electron density changes with altitude, the latitudinal density gradient more or less remains the same at all altitudes. Therefore, the density gradient derived in terms of the normalized EDF at a given ionospheric altitude may reflect the gradient for other altitudes also. However, during geomagnetic storms the ionospheric – plasmaspheric density changes significantly thus this assumption requires extra caution^30–32^.

Next, we compute the SGF using Eq. (4). The GPS satellites orbit in near-circular paths at ~22,000 km. On account of such high orbital elevation and very less eccentricity (<0.02), the satellite azimuth-elevation angles (i.e. LOS) vary in the same manner at any geographical location at a given time. However, the orientation of satellite LOS with seismo-acoustic rays vary with respect to the station-source location. Thus, the SGF has to be estimated at each ground station adjoining the source. The orientation of a satellite LOS with vertically propagating seismo-acoustic waves can have two extremes. The schematic in Fig. S4 shows realistic interaction between the vertically propagating seismo-acoustic rays and satellite LOS in 2D for station located at 400 km from the Trial source. If both vectors (LOS and wave) are orientated at angles ≅ 180° then the absolute values of SGF ≅ 1 (Eq. 3) and the perturbation phases integrate the CIP amplitudes to zero (case-II in Fig. S4). The favorable satellite LOS geometries occur at orientations near 90° with respect to the wave vector (case-I in Fig. S4) and the SGF values ≅ 0 on the occasions (Eq. 3).

It is noteworthy that the maximum favorable GNF value is 1 while the SGF favors most at 0. This flip-flop behavior is due to the geometry of the respective vectors involved in the computation of these factors. Since we aim to combine both these factors and to propose a single model estimation of non-tectonic forcing mechanisms surrounding a seismic source, the current mathematical expressions of these two factors may not be adequate. Thus, we transform the algebraic expression for SGF to Eq. (4) which computes variations closer to 1 for favorable satellite geometry.

As mentioned, the SGF has to be computed for individual GNSS stations. We assume three stations located at 2°, 4° and 6° south of the Trial source and compute SGF for all possible satellite LOS from a specific station (Fig. 2). From Fig. 2, for a station situated near to the Trial epicenter (200 km), the GNSS satellite geometry moderately supports the evolution of CIP. The SGF gradually becomes favorable as the station-source distance increases. It should be noted that the SGF computation would manifest in the same fashion for a station located in the north, east, and west. The maximum station-source distance is restricted within 6° (~600 km) as beyond this distance, the seismo-acoustic rays start refracting downward from ionospheric peak density altitude of 350 km. Further, since ionospheric peak density altitude is 350 km in the Trial case, we could consider the station till 6°. However, in the case of lower ionospheric peak density altitude, the station has to be closer than this.

In the final step, we estimate the NTFM factor at an ionospheric altitude of 350 km using Eq. (5) and present in Fig. 2. We segregate the model output in three domains: (i) [1 0.5] as highly favorable to favorable, (ii) [0.5 0.3] as favorable to moderately favorable (iii) [0.3 0] as moderately favorable to poor. The NTFM factor, in Fig. 2, suggests that the evolution of ionospheric perturbations is favored equatorward, especially for GPS stations located at 4° to 6° latitudinal distance from the source. The CIP growth might be hampered towards the north mainly due to the poor GNF and SGF. Further, for a station located near to the source (2°), the CIP evolution towards the equator might be moderate due to the moderate SGF. It should be noted that Fig. 2 presents the modeled NTFM factor along with GNF, EDF, and SGF at a single altitude of 350 km.

**Figure 3:**
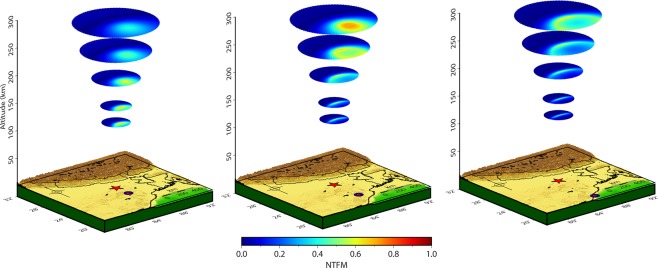
3D manifestation of NTFM factor for the Trial source. The figure is prepared using the GMT 5.4.4^43^.

We extend this analysis for various atmospheric altitudes, from 120 km to 300 km, and present the modeled 3D NTFM factor in Fig. 3. It should be noted that ionospheric altitudes of ~110–120 km and above can only host the ionospheric perturbations due to the fact that ionization density is very small below these altitudes. Therefore, the NTFM factor computation is started at 120 km altitude. The next atmospheric altitude is considered at 150 km and then at every 50 km, the factor is computed for stations located at 2°, 4°, and 6° latitudes from the Trial source (Fig. 3). In Fig. 3, for a station located close to the source (at 2°) the non-tectonic effects are more favorable at lower ionospheric altitudes than the higher ones. Whilst, for a station located at 4° the NTFM factor favors the evolution of ionospheric perturbations at higher ionospheric altitudes. The favorable NTFM factor gradually weakens as the station distance increases beyond 4° from the source. This could be verified from a station located at 6° latitude.

It is important to note that the NTFM factor and their horizontal extent vary significantly with altitudes. The variations in the horizontal extent are attributed to the atmospheric refraction of seismo-acoustic rays (Figs. 1, S3 and S4). Since the GNF and SGF computations are based on the propagation of seismo-acoustic rays, the horizontal extent of the NTFM model output evidently reflects the wave propagation characteristics at various atmospheric altitudes. Further, the altitudinal variations of NTFM factor (i.e. favorable and vice versa) depend on the manifestations of GNF and SGF. The geomagnetic field inclination and declination do not vary significantly with altitudes. But the propagation angles of seismo-acoustic rays vary considerably. Therefore, GNF computed based on these two parameters (Eq. 2) varies with altitudes. For SGF, seismo-acoustic wave vectors, satellite LOS, and station-source distance are the key parameters. In the Trial case, for a station located at 2°, the favorable satellite line of sights happen to be those with higher elevations (83°–90°) that align suitably with the upward propagating seismo-acoustic rays (parallel to wavefronts) and thus favor the evolution of CIP. For stations located at 4° and 6°, the favorable line of sights occur for elevation range of ~46° to ~90° (Fig. S4) and ~33° to ~90° respectively. The varying alignments of such satellite geometries with vertically propagating seismo-acoustic rays significantly control the altitudinal behavior of NTFM factor through SGF.

### Validation

In this section, we validate our proposed NTFM model based on the near field coseismic ionospheric response to actual earthquake events that occurred at different latitudes. For this, we analyze ionospheric variations during the Mw 8.2 01 April 2014 Iquique (IQ henceforth) earthquake, Mw 8.3 16 September 2015 Illapel (IL henceforth) earthquake, Mw 7.8 25 April 2015 Nepal (NP henceforth) earthquake, Mw 8.6, 11 April 2012 Sumatra (SU henceforth) earthquake, and Mw 7.8 13 November 2016 New Zealand (NZ henceforth) earthquake. It should be noted that all these earthquakes occurred on geomagnetically quiescent days.

The IQ and IL earthquakes triggered near the Chile subduction zone. The seismicity over the Chilean region is defined by the subduction of Nazca plate under the South American plate at the Peru-Chile trench. This subduction zone is well known for hosting the great and giant megathrust earthquakes^33^. The IQ and IL earthquakes are examples of this. The IQ earthquake ruptured the center part of the northern Chile seismic gap (~18°S–24°S)^34^ with hypocenter at 19.61°S 70.77°W at a depth of ~25 km (https://earthquake.usgs.gov). Extensive studies on source characteristics, constrained by various slip models and data suggest that the seismic rupture propagated south of the epicenter during this offshore event^34–38^.

**Figure 4:**
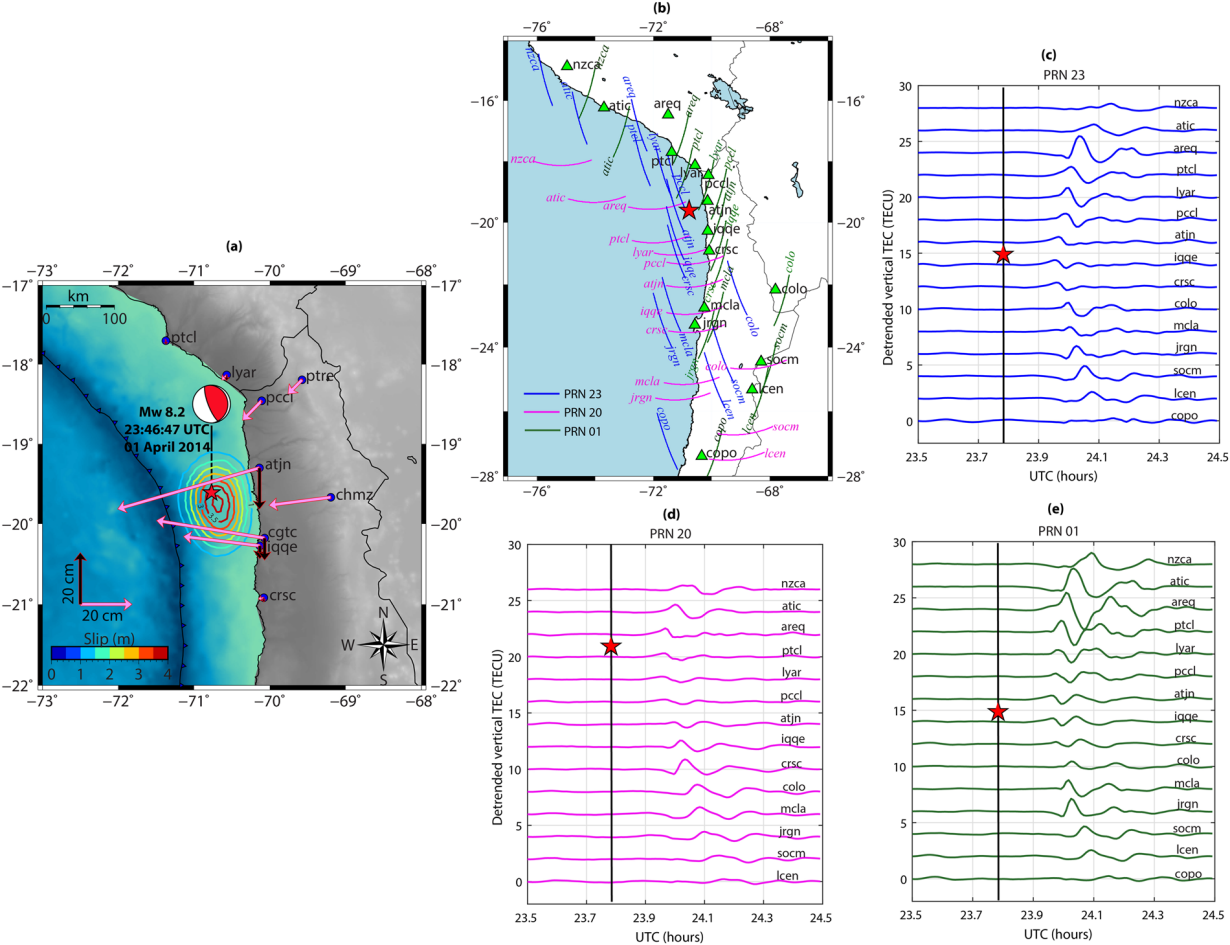
(**a**) Geographical map showing the epicenter location and the fault mechanism during the Mw 8.2 01 April 2014 Iquique (IQ) earthquake. The arrows demonstrate the horizontal and vertical displacement fields computed in this study. The colored contours show the slip distribution during the event and are taken from Geersen *et al*.^34^. The figure is prepared using the GMT 5.4.4^43^. (**b**) IPP tracks of PRNs 23, 01, and 20 at peak density altitude of 350 km during the earthquake occurrence period. The peak density altitude is derived based on the IRI-2016 model. The tracks are labeled with their respective observing station names. The locations of stations are shown with triangles. (**c–e**) Temporal evolution of CIP as observed respectively by PRNs 23, 01, and 20. Each time series is labeled with respective recording station names. The red star in each panel shows the epicenter location for easier understanding of CIP evolution north and south of the epicenter. The vertical lines in (**c–e**) show the earthquake onset time.

From a geomagnetic coordinate point of view, the IQ earthquake could be categorized as a *near-equatorial earthquake* (~−15.16° magnetic inclination). Figure S2 could be referred for more information on this. The geographical map in Fig. 4a shows the location of IQ epicenter and the earthquake fault mechanism (thrust). The figure also contains the static horizontal and vertical displacement fields estimated using nearby GPS geodetic observations. The displacement fields, presented with horizontal and vertical arrows, exhibited profound westward and downward movements at the coast. From the figure, the maximum of westward and downward movements respectively of ~57.08 cm and ~15.91 cm were observed at *atjn* station. Further, GPS stations from the south of the epicenter also observed significant displacements. The slip variations along with the estimated displacement fields corroborate the maximum energy propagation south of the epicenter. Therefore, the coseismic ionospheric response should be more intense in the south during this event.

Figure 4b–e demonstrate the ionospheric variations over the IQ source region. GPS satellites with Pseudo Random Noise (PRN) codes 01, 20, and 23 from stations shown in Fig. 4b were found to be suitable to observe the near field CIP. This could be verified from the IPP tracks of these satellites in Fig. 4b. The satellites adequately cover the ionosphere over and around the IQ earthquake fault region. Figure 4c–e show the temporal evolution of CIP as observed by these PRNs. The CIP are derived as detrended vertical TEC. Each time series is presented with same color as that of the respective satellite track. PRN 23 and 01 captured clear and intense CIP far north and south of the epicenter. However, the satellites recorded relatively feeble CIP closer to the epicenter. Interestingly, the CIP as recorded by PRN 01 from far north stations of *areq*, *atic* and *nzca* were more intense. In contrast to these, PRN 20 recorded more CIP amplitudes south of the epicenter. Due to the fact that most of the seismic energy propagated south of the epicenter, the CIP should evolve stronger in the south compared to those in the north. However, maxima of CIP amplitudes, as recorded by PRN 23 and PRN 01 were observed far north of the epicenter.

**Figure 5:**
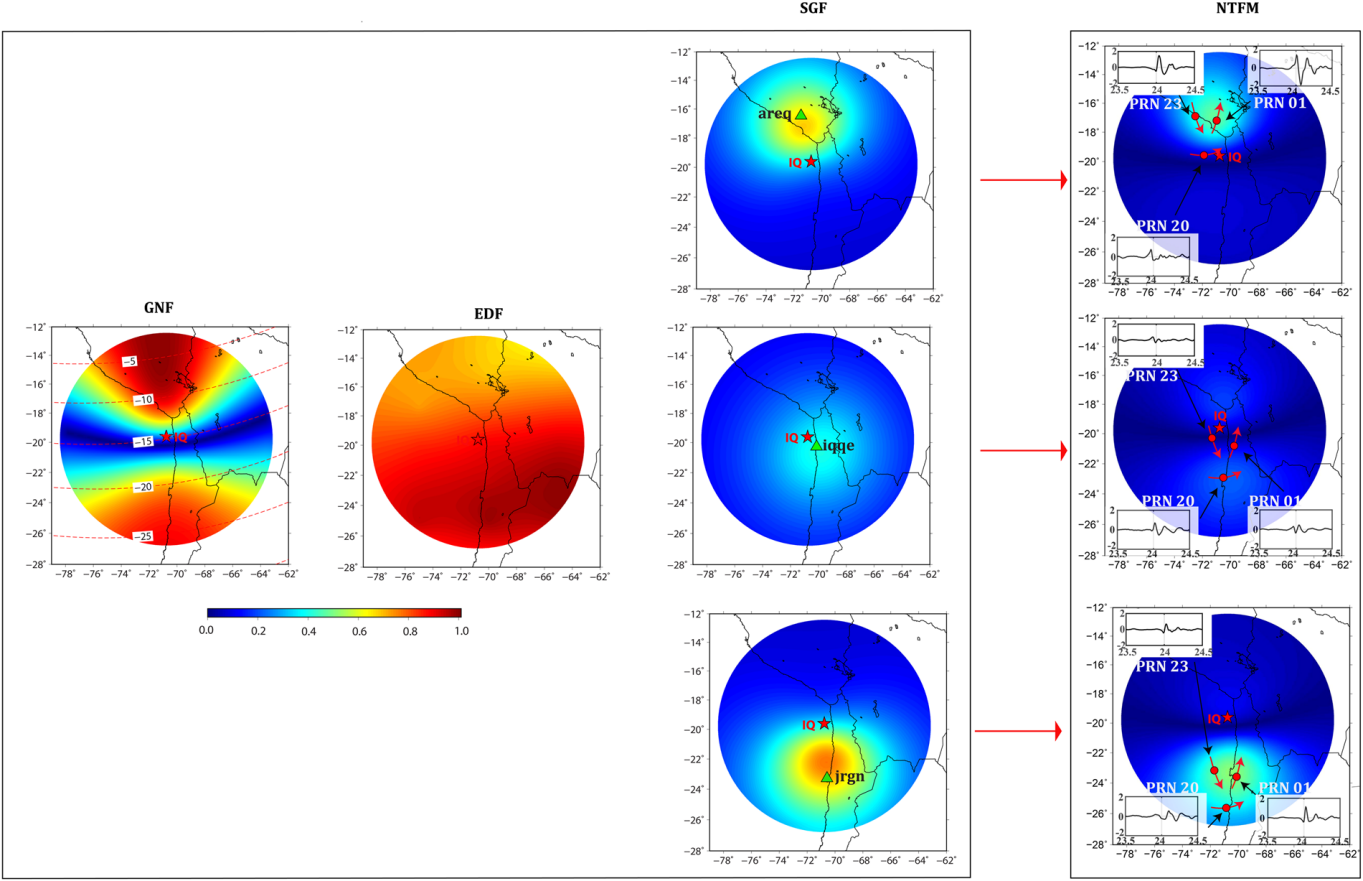
2D manifestation of NTFM model in terms of GNF, EDF, SGF, and NTFM factor at IPP altitude of 350 km during the IQ earthquake. The SGF is estimated for GPS stations of *iqqe, jrgn*, and *areq*. These stations are located at the epicentral distance of ~99 km, ~409 km, and ~358 km respectively. The figure is prepared using the GMT 5.4.4^43^.

We attempt to quantify the captured ionospheric imprints of seismic energy during the IQ event in light of the manifestation of non-tectonic forcing mechanisms surrounding the earthquake source region. We run the proposed 3D NTFM model to compute the GNF, EDF, SGF and their combined effects as NTFM factor and present the model output at IPP altitude of 350 km in Fig. 5. The red star demonstrates the ionospheric projection of the IQ epicenter. From the estimated GNF variations, the geomagnetic field favored the CIP evolution more in the north as compared to the south during the IQ earthquake. Moreover, the region far north was relatively more favorable for the evolution of CIP from the geomagnetic field geometry point of view. The EDF exhibited relatively higher density in the south during the earthquake occurrence time. The modelled SGF variations in Fig. 5 suggest that the GPS satellite geometry from stations closer to the IQ epicenter (200 km and less) obstruct the CIP evolution. In contrast to this, the SGF considerably favors the CIP evolution for stations located between 200–400 km, both north and south of the epicenter. Finally, the combined NTFM factor in Fig. 5 suggests that stations located within 200 km of the IQ epicenter were not adequate to capture the seismic energy imprints during the event. However, distant stations could capture this energy in a more profound manner.

We assume that poor manifestations of NTFM factor for the nearby GNSS stations (<200 km) did not allow the maximum released seismic energy to evolve in the ionosphere. But in the north, CIP amplitudes remained higher due to the favorable non-tectonic forcing mechanisms. Significant CIP amplitudes were observed in *areq-PRN 01* and *areq-PRN 23*. From Fig. 5, the NTFM factor exhibited favorable to moderately favorable domain for the evolution of CIP at the IPP locations of these PRNs. Near the IQ epicenter, comparatively lesser CIP amplitudes were observed by *iqqe-PRN 01*, *iqqe-PRN 20*, and *iqqe-PRN 23* which are in accordance with the poor NTFM factor. However, irrespective of a similar or more favorable non-tectonic forcing mechanisms, CIP amplitudes remained smaller from *jrgn* station than those of *areq*. This could be attributed to the larger distance between the ionospheric projection of the IQ epicenter and the IPPs from *jrgn* station. Thus, our proposed 3D NTFM model, in light of the prevailing non-tectonic forcing mechanisms, effectively explains the ionospheric variations induced by the earthquake nucleating at near-equatorial latitude.

**Figure 6:**
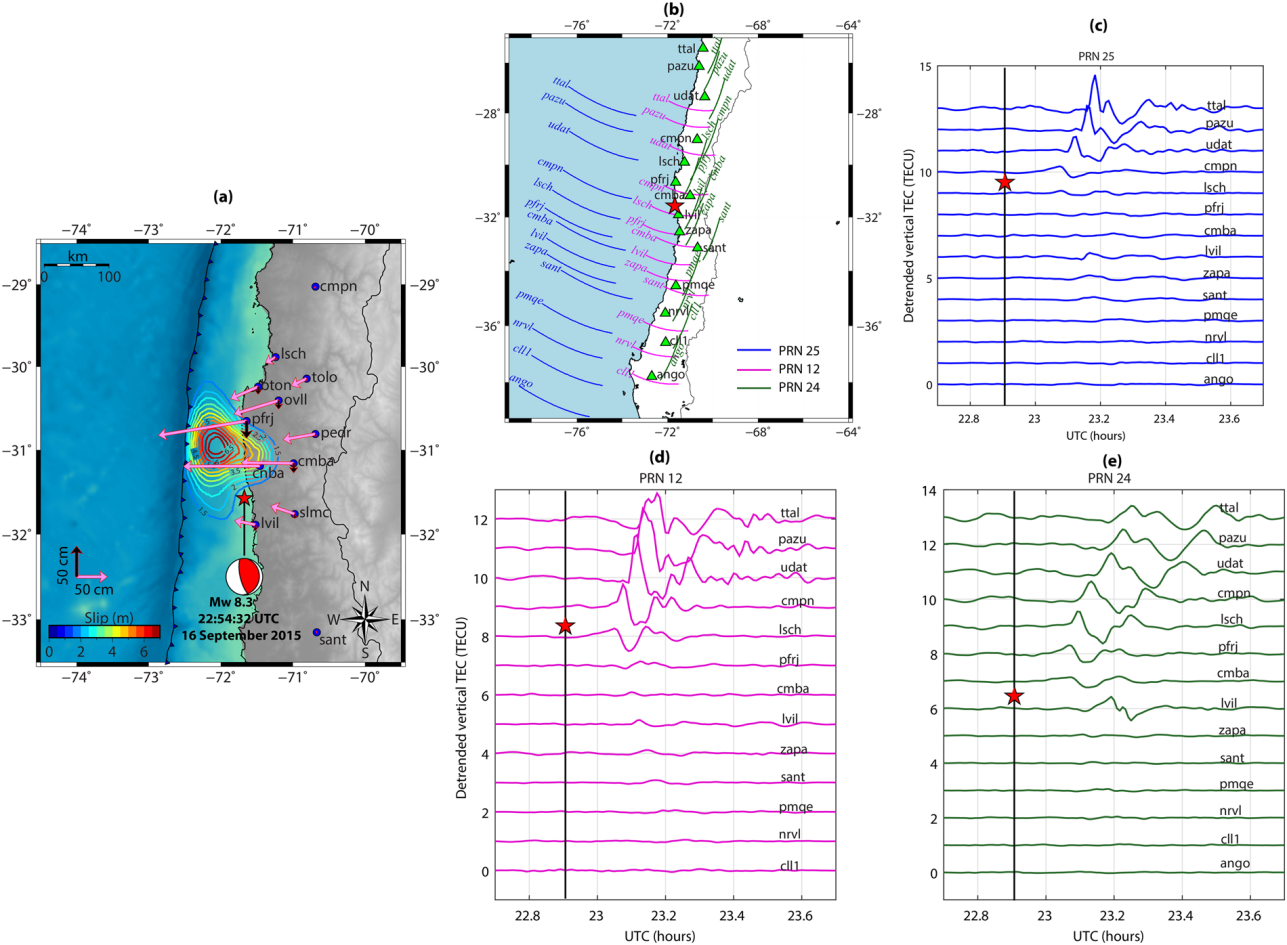
(**a**) Epicenter location and fault mechanism during the Mw 8.3 16 September 2015 Illaple (IL) earthquake. The estimated horizontal and vertical displacement fields are presented with arrows. The color contours show the slip distribution adopted from Srivastava *et al*.^39^. The figure is prepared using the GMT 5.4.4^43^. (**b**) IPP tracks of PRNs 25, 12, and 24 at ionospheric peak density altitude of 350 km during the IL earthquake period. The peak density altitude information is derived based on the IRI-2016 model. (**c–e**) Temporal evolution of CIP as observed by PRNs 25, 12, and 24 respectively. Other details are same as those in Fig. 4(b–e).

The IL earthquake is another great subduction earthquake that ruptured the offshore region of central Chile with a hypocenter located at 31.57°S 71.67°W at a depth of ~22.3 km (https://earthquake.usgs.gov). The geographical map in Fig. 6a shows the IL earthquake epicenter location, fault mechanism (thrust) and static horizontal and vertical displacement fields estimated using the geodetic observations from nearby GPS sites. From the figure, significant trenchward (westward) horizontal displacement is evident at all GPS sites. The maximum horizontal displacement of ~1.43 m was observed at *pfrj* GPS station. A vertical uplift of ~2.67 cm was recorded at *cnba* GPS site while other GPS sites subsided by few millimeters to centimeters. This suggests that the offshore coseismic slip exhibited significant slip distribution below the *pfrj* and *cnba* GPS stations^39^. From the estimated displacement fields and reproduced slip distribution, it is obvious that most of the seismic moment was released north of the epicenter during this event.

The IL earthquake triggered at the geomagnetic low latitude region. The magnetic inclination at the epicenter location is ~−32.43°. This could be verified from Fig. S2. Thus, we classify this earthquake as a *low latitude earthquake*. The TEC as observed by PRNs 25, 12, and 24 from GPS stations shown in Fig. 6b were scrutinized into for coseismic ionospheric variations during the IL event. The IPP trajectories of these PRNs at the earthquake occurrence time are also shown in the figure. From Fig. 6c–e, all three PRNs could capture discernible CIP surrounding the epicenter. However, higher CIP amplitudes were observed north of the epicenter.

**Figure 7:**
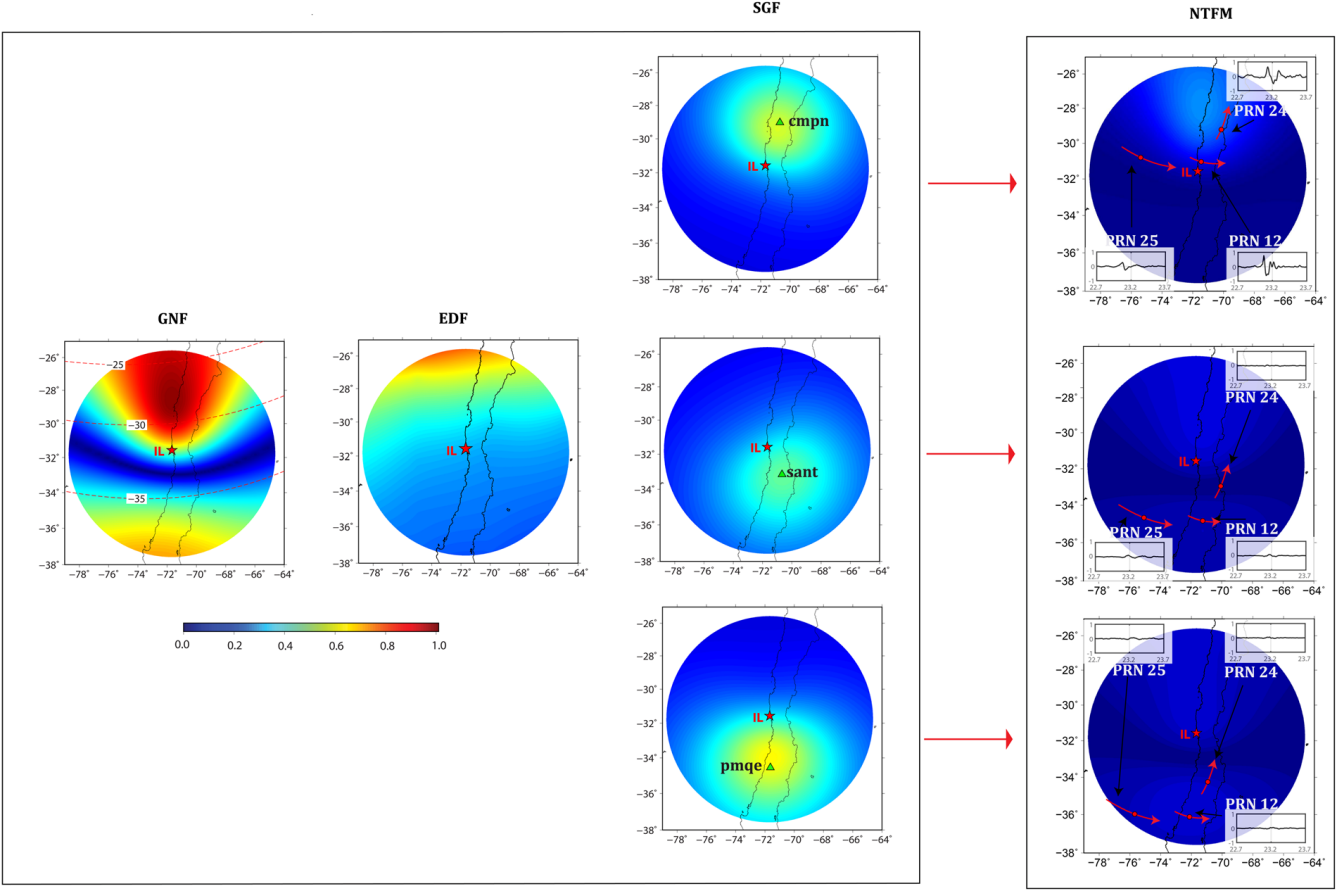
2D manifestation of NTFM model in terms of GNF, EDF, SGF, and NTFM factor at IPP altitude of 350 km during the IL earthquake. The SGF is estimated for *cmpn*, *sant*, and *pmqe* stations located respectively at ~298 km, ~199 km, and ~331 km from the IL epicenter. The figure is prepared using the GMT 5.4.4^43^.

To quantify the effects of non-tectonic forcing mechanisms on the observed CIP manifestations, we run our 3D NTFM model for the earthquake source located in low latitude region. The model output, estimated at IPP altitude of 350 km, in terms of GNF, EDF, SGF, and NTFM factor are presented in Fig. 7. The GNF behavior demonstrates that the geomagnetic field favored the CIP evolution more in the north during the IL earthquake. Also the ambient electron density gradient was higher towards the north. The modelled SGF variations corroborate the earlier results for Trial and IQ earthquakes and suggest that GPS stations located within ~200–400 km of epicenter are more suitable to observe the seismic energy imprints in the ionosphere. The estimated NTFM factors for *cmpn*, *sant* and *pmqe* stations indicate that CIP evolution in the north was highly favored by the non-tectonic forcing mechanisms. However, the CIP could not develop in the south mainly due to the poor GNF and EDF. More clarity on this could be obtained by examining the NTFM factor manifestation along the respective satellite tracks from *cmpn*, *sant* and *pmqe* GPS stations in Fig. 7. From Figs. 6 and 7, the CIP evolution preferentially follows the seismic energy propagation during the IL event.

**Figure 8:**
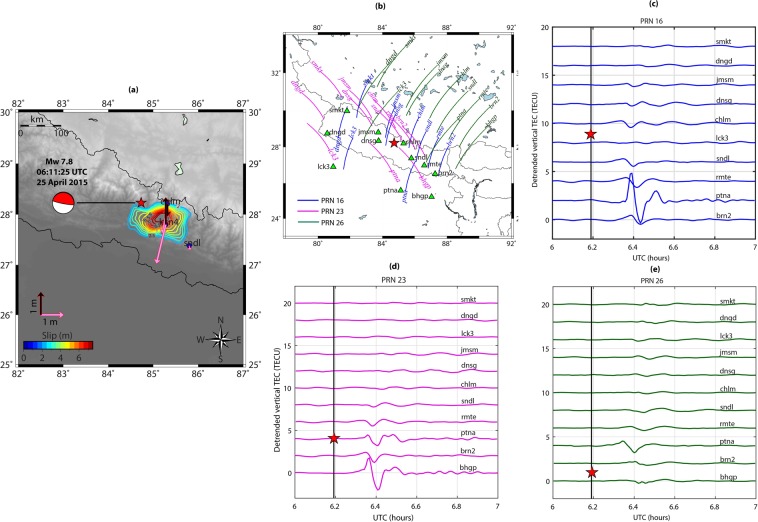
(**a**) Topography map showing the epicenter location and fault mechanism during the Mw 7.8 25 April 2015 Nepal (NP) earthquake. The computed horizontal and vertical displacement fields are presented with arrows. The colored contours demonstrate slip distribution taken from Yagi and Okuwari, 2015^44^. The figure is prepared using the GMT 5.4.4^43^. (**b**) IPP tracks of PRNs 16, 26, and 23 at ionospheric peak density altitude of 350 km during the earthquake period. The peak electron density altitude is extracted from IRI-2016 model. (**c–e**) Evolution of coseismic ionospheric perturbations along the tracks of PRNs 16, 26, and 23 respectively. Other details are same as those in Fig. 4 (**b–e**).

The NP earthquake ruptured the Main Himalayan Thrust (MHT) and was the largest earthquake to occur in the central Himalayas since the 1934 Bihar-Nepal earthquake. The earthquake nucleated at 28.23°N 84.73°E and at a depth of ~8.2 km on 25 April 2015 at ~06:11 UT (https://earthquake.usgs.gov). Figure 8a shows the location of the NP epicenter and the fault mechanism (thrust). The arrows demonstrate the static horizontal and vertical displacement fields estimated using nearby GPS geodetic observations. The horizontal movement of **~**1.75 m in the south-southwest and uplift of ~1.17 m were observed at *kkn4* station. The *chlm* GPS station subsided by ~0.54 m. From Fig. 8a, most of the seismic energy during the NP earthquake propagated in the east-southeast of the epicenter^5,9^.

Based on the magnetic inclination variations (Fig. S2), the NP earthquake could be categorized as *low-mid latitude* event (magnetic inclination ~44.31). From Fig. 8b–e, GPS satellites with PRN codes 16, 23, and 26 were rightly located over the NP fault region during the earthquake period and could capture significant ionospheric signatures from the GPS stations located surrounding the epicenter. It should be noted that PRNs 16, 23, and 26 observed significant CIP amplitudes southeast of the epicenter. IPP tracks of these PRNs in Fig. 8b and temporal evolution of CIP as recorded by respective PRNs in Fig. 8(c–e), specially CIP time series of *ptna-16*, *bhgp-23*, and *ptna-23*, could be verified for this. However, the CIP that observed north and southwest of the epicenter could not exhibit comparable amplitudes.

**Figure 9:**
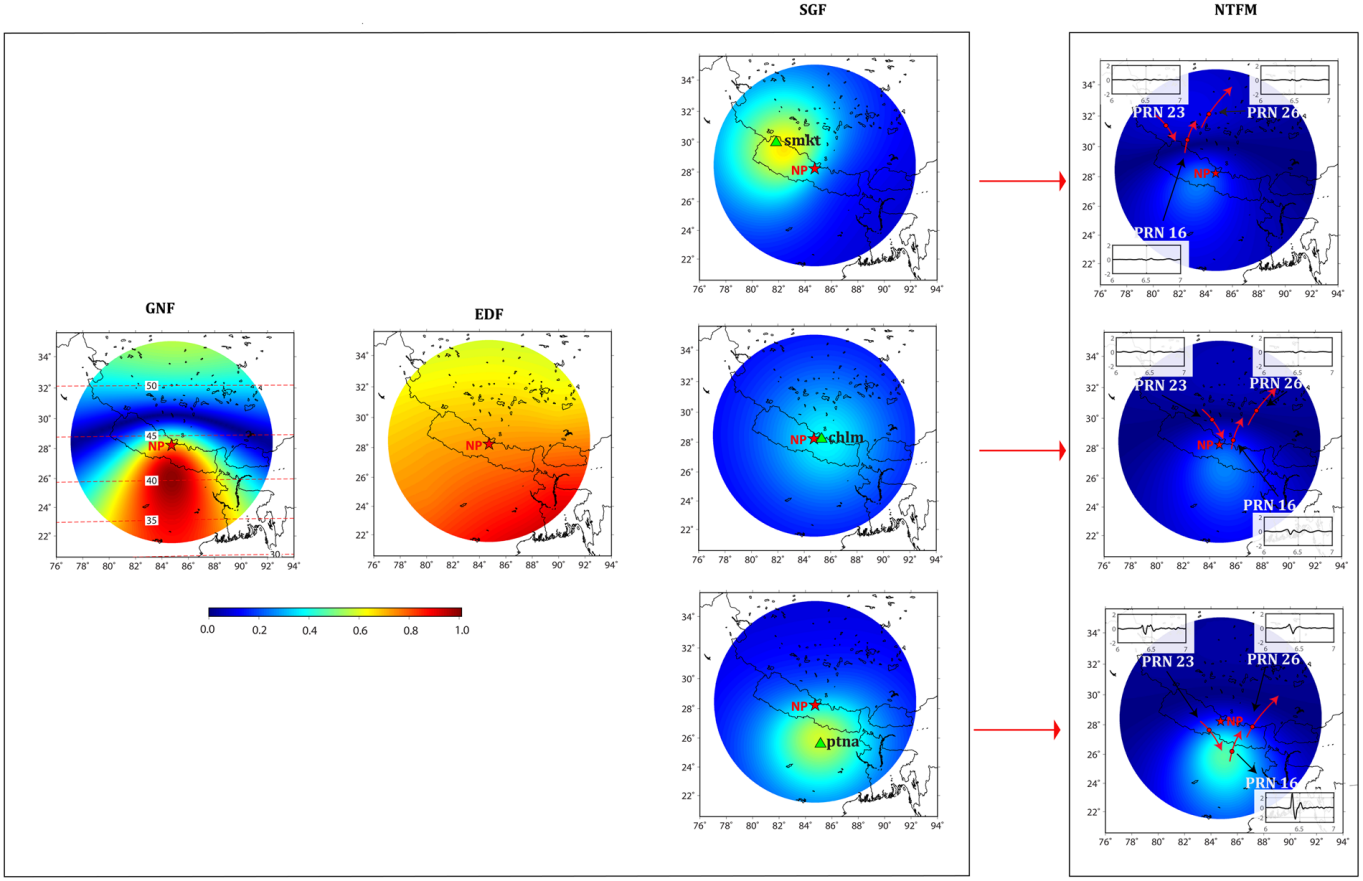
2D manifestation of GNF, EDF, SGF, and their collective effects as NTFM factor at IPP altitude of 350 km surrounding the NP earthquake epicenter. The SGF is estimated at GPS stations of *chlm*, *smkt*, and *ptna* located at respective distances of ~50 km, ~195 km, and ~300 km from the epicenter. The figure is prepared using the GMT 5.4.4^43^.

The observed coseismic ionospheric signatures during the NP earthquake are then examined for the effects of non-tectonic forcing mechanisms. We estimate the GNF, EDF, SGF, and total effects of all three based on the proposed model and present the output at IPP altitude of 350 km in Fig. 9. The GNF favors the CIP evolution south of the NP epicenter i.e. equatorward and impedes the evolution in the north. The GNF estimated during the NP earthquake corroborated well with that of the Trial source. From the estimated EDF over the NP seismic source, the electron density was higher in the southeast of the epicenter. For SGF estimation, we select GPS stations of *chlm*, *smkt*, and *ptna*. The modelled SGF was favorable for the manifestation of ionospheric signatures from stations located at the epicentral distance of ~200 km and beyond (i.e. *smkt* and *ptna*). However, the SGF was moderate to poor from a station closer than this. The estimated SGF also corroborated well with that of the Trial source. The NTFM model output in Fig. 9 demonstrates that non-tectonic forcing mechanisms favored the CIP evolution south of the epicenter during the NP earthquake. Whilst, the CIP in the north were not supported by these mechanisms. Therefore, for a source located in the northern low latitudes, if seismic energy propagates south of the epicenter (equatorward) then the induced ionospheric perturbations could be well captured by the stations located south of the seismic source. It should be noted that though the NTFM factor was favorable south of the NP epicenter, the CIP preferably evolved in the southeast which substantiate the seismic rupture propagation in the same direction.

We extend a similar analysis during the SU (near-equatorial latitude), and NZ (mid latitude) earthquakes. The SU earthquake was one of the largest strike-slip events recorded ever. The rupture process of this earthquake was reported to be quite complex with a sequence of ruptures propagating westward to the epicenter^40,41^. The strike-slip faults that ruptured were mainly trending west-northwest to east-southeast and north-northwest to south-southwest. Figure S5 shows the coseismic ionospheric response to this event as observed by PRNs 32, 16, and 06 from various Sumatran GPS Array (SuGAR) stations. It could be noted that PRNs orbiting north and south of the epicenter recorded discernible CIP. However, CIP remained quite feeble nearby the epicenter (e.g. *umlh-6, bthl-16*). The NTFM factor along with GNF, EDF, and SGF during the SU event is shown in Fig. S6. Similar to the IQ earthquake, the NTFM factor during the SU event favored the CIP evolution in the north and south of the epicenter and hampered the same nearby the epicenter. Interestingly, the CIP amplitudes were consistently larger towards the north. This corroborated with the fact that maximum vertical displacement during this event was observed in the north of the epicenter^4,42^. Despite the complex rupture process during the SU event, the coseismic ionospheric response could reflect the maximum tectonic energy propagation reasonably well.

Bagiya *et al*.^7^ analyzed the NZ earthquake and explained the coseismic ionospheric response based on the proposition of tectonic thrust orientations as distinct sources responsible for peculiar CIP azimuthal propagation surrounding the Kaikoura epicenter. This novel proposition was derived considering the non-tectonic forcing mechanisms in the account. However, they could not propose any mechanism to quantify all three non-tectonic effects collectively. It may be recalled that NZ earthquake was a mid latitude event. We reanalyzed this event using our proposed model and considering the Campbell Coseismic Thrust Zone as the source of CIP^7^. We found that during the NZ event also the non-tectonic forcing mechanisms support the equatorward evolution of seismic induced ionospheric perturbations. This could be realized from the evolution of CIP over the NZ fault region presented in Fig. S7. The NTFM model output during the NZ earthquake is presented in Fig. S8 to acquire better clarity on this.

**Figure 10:**
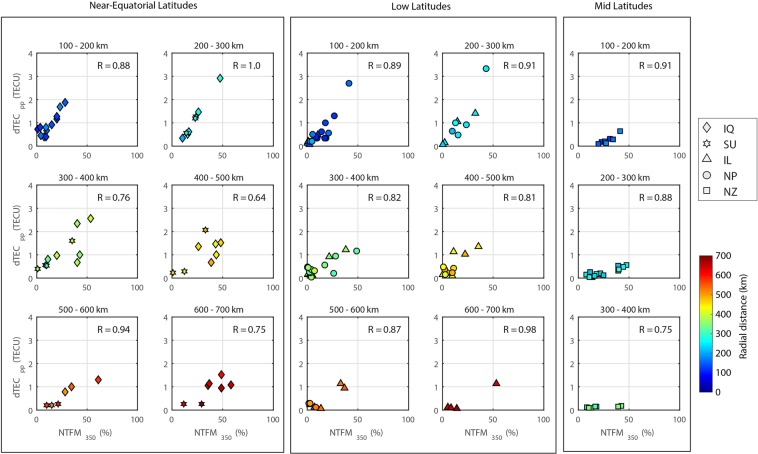
Correlation between the peak-to-peak CIP amplitude variations and corresponding values of NTFM factor computed at respective IPP altitudes for near-equatorial, low and mid latitude earthquakes. The correlation is estimated at radial distances of (**a**) 100–200 km (**b**) 200–300 km (**c**) 300–400 km (**d**) 400–500 km (**e**) 500–600 km and (**f**) 600–700 km from ionospheric projection of respective epicenters. R is the correlation coefficient and computed based on the linear regression fit to the data. The NTFM factor variations are presented in percentage. The symbol color represents the distance of individual CIP from the ionospheric projection of the respective epicenter. IQ, SU, IL, NP, and NZ denote Iquique, Sumatra, Illapel, Nepal, and New Zealand earthquakes, respectively.

In the final step, we evaluate the correlation between the peak-to-peak amplitude variations of CIP and corresponding variations of NTFM factor at IPP altitude of 350 km for each satellite and station pairs shown in Figs. 4, 6, 8, S5 and S7 and present in Fig. 10. The correlation analysis is presented for different latitude conditions. Therefore, the figure contains three sets of events analyzed in this case study i.e. near-equatorial, low, and mid latitudes. The region around the ionospheric projection of respective epicenters is divided into various concentric circles with a 100 km span. The correlation is estimated at each range from a radial distance of 100 km and onward. The ionospheric perturbations corresponding to each event are presented with a specific symbol for easy classification.

It could be noted that the correlation between CIP amplitudes and corresponding NTFM factor withstands fairly well at each range of distance. However, the amplitudes weaken beyond 400 km of radial distance in the present case study. We propose that beyond these threshold distances, direct propagation of epicentral energy may not be adequate to induce significant ionospheric signatures even if the NTFM factor is favorable. This allows us to provide a first estimation of the threshold distance for the CIP evolution from the viewpoint of direct epicentral energy propagation and the impact of non-tectonic forcing around the epicenter. The correlation analysis for the radial distance <100 km was not feasible due to a lack of sufficient CIP observations. Further, during the NZ earthquake event, very few CIP were observed after 400 km so no correlation analysis is presented.

From the present study, it should be noted that for earthquakes occurring at near-equatorial latitudes, the NTFM factor favors the evolution of CIP north and south of the epicenter (>200 km) and not nearby the epicenter. The coseismic ionospheric variations during the IQ and SU earthquakes could be verified for this. Further, for earthquake nucleating at low and mid latitudes, the NTFM factor favors the CIP manifestation equatorward. The coseismic ionospheric responses to the NP, IL and NZ earthquakes substantiate this. For easier and quick review of this study, the response of NTFM model for earthquakes occurring at different latitudes is summarized in Table 1.

**Table 1:** Review on the manifestation of proposed 3D NTFM model for seismic sources located at various latitudes.

Epicenter location (in geomagnetic coordinates)	Favorable regime of non- tectonic forcing mechanisms	Unfavorable regime of non- tectonic forcing mechanisms
Magnetic inclination = 0°	North and South of the epicenter	Epicenter and nearby region
Magnetic inclination ≥ 0° and ≤ ~ 40° (low latitude)	Equatorward	Poleward (i.e. north or south)
Magnetic inclination ≥ ~ 40° and ≤ ~ 60° (mid latitude)	Equatorward	Poleward (i.e. north or south)

## Conclusion

A 3D model to map the combined effects of the non-tectonic forcing mechanisms of geomagnetic field, GNSS satellite geometry, and ambient electron density gradient is proposed for the first time. The 3D NTFM model can compute these effects at various ionospheric altitudes depending on the propagation characteristics of seismo-acoustic rays. The model not only successfully explains the ionospheric manifestations during seismic events occurring at different latitudes but also cautions that any correlation between the seismic source manifestations at the ground and corresponding ionospheric perturbations could be erroneous in the absence of quantifying the effects of these mechanisms. Further, the threshold distance from the viewpoint of direct epicentral energy propagation and impact of non-tectonic forcing around the epicenter is modeled for the first time. It should be noted that the proposed 3D model is specifically designed for the spatial analysis of GPS-TEC derived seismic induced ionospheric perturbations.

The preparation of GUI based online version of the proposed model is in progress to facilitate the researcher to quantify the non-tectonic effects on ionospheric perturbations during earthquakes occurring at various latitudes. This model will be shortly online at iigm.res.in. It is believed that the proposed study will assist the researchers to identify the non-tectonic effects on the GNSS measured CIP without performing any further quantitative analysis.

## Data Availability

GPS-TEC data over the Chile and Nepal regions are obtained respectively from http://gps.csn.uchile.cl/ and https://www.unavco.org/data/.

## Supporting Information

Supporting Information
