# Supplementary material for: Mapping the Impact of Non-Tectonic Forcing mechanisms on GNSS measured Coseismic Ionospheric Perturbations
Source: Sci Rep. 2019 Dec 9;9:18640. doi: 10.1038/s41598-019-54354-0 (PMC6901456; doi:10.1038/s41598-019-54354-0)
Supplement: Supplementary file 1 — Supporting Information [file 41598_2019_54354_MOESM1_ESM.doc]

**Supplementary Information**

**Mapping the Impact of Non-Tectonic Forcing mechanisms on GNSS measured Coseismic Ionospheric Perturbations**

Mala S. Bagiya^1^, A. S. Sunil^1^, Lucie Rolland^2^, Srinivas Nayak^1^, M. Ponraj^1^, Dhanya Thomas^1^ and D. S. Ramesh^1^

^1^Indian Institute of Geomagnetism (DST), Navi Mumbai, India

^2^Université Côte d’Azur, OCA, CNRS, IRD, Géoazur, Sophia-Antipolis, Valbonne, France

*Corresponding Author: [bagiyamala@gmail.com](mailto:bagiyamala@gmail.com)

**Supplementary**

**Figures S1, S2, S3, S4, S5, S6, S7, and S8**

**
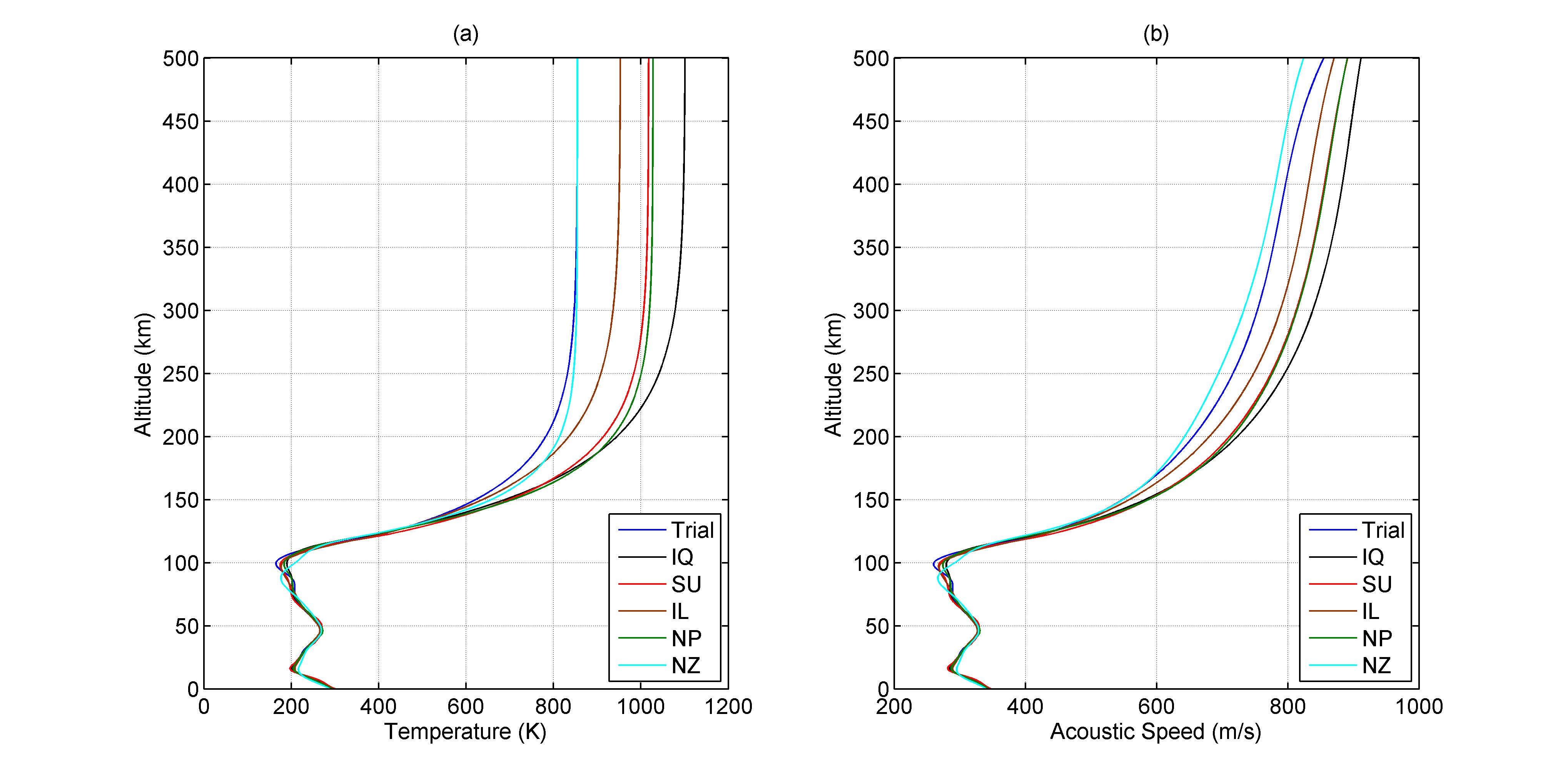
**

**­­Figure S1:** (a) Atmospheric temperature variations derived from the NRLMSISE-00 model at the occurrence time of Trial, IQ, SU, IL, NP, and NZ earthquakes (b) Acoustic wave velocity variations with atmospheric altitudes estimated based on the respective temperature profiles shown in (a).

**
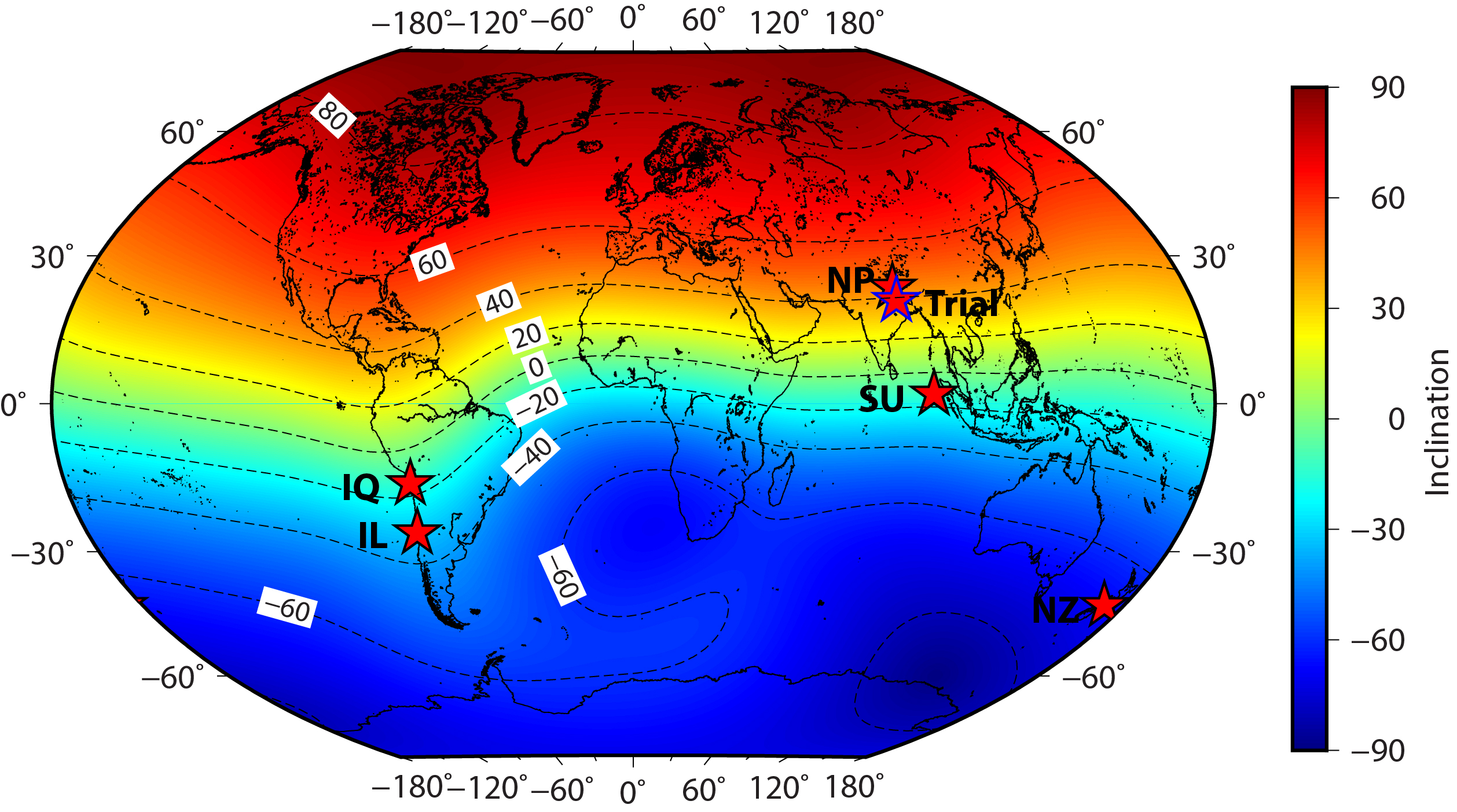
**

**Figure S2:** Global map of magnetic inclination (dip) variations based on the values provided by the IGRF-12 model. Locations of Trial, IQ, SU, IL, NP, and NZ epicenters are shown with stars. The figure is prepared using the GMT 5.4.4^43^.


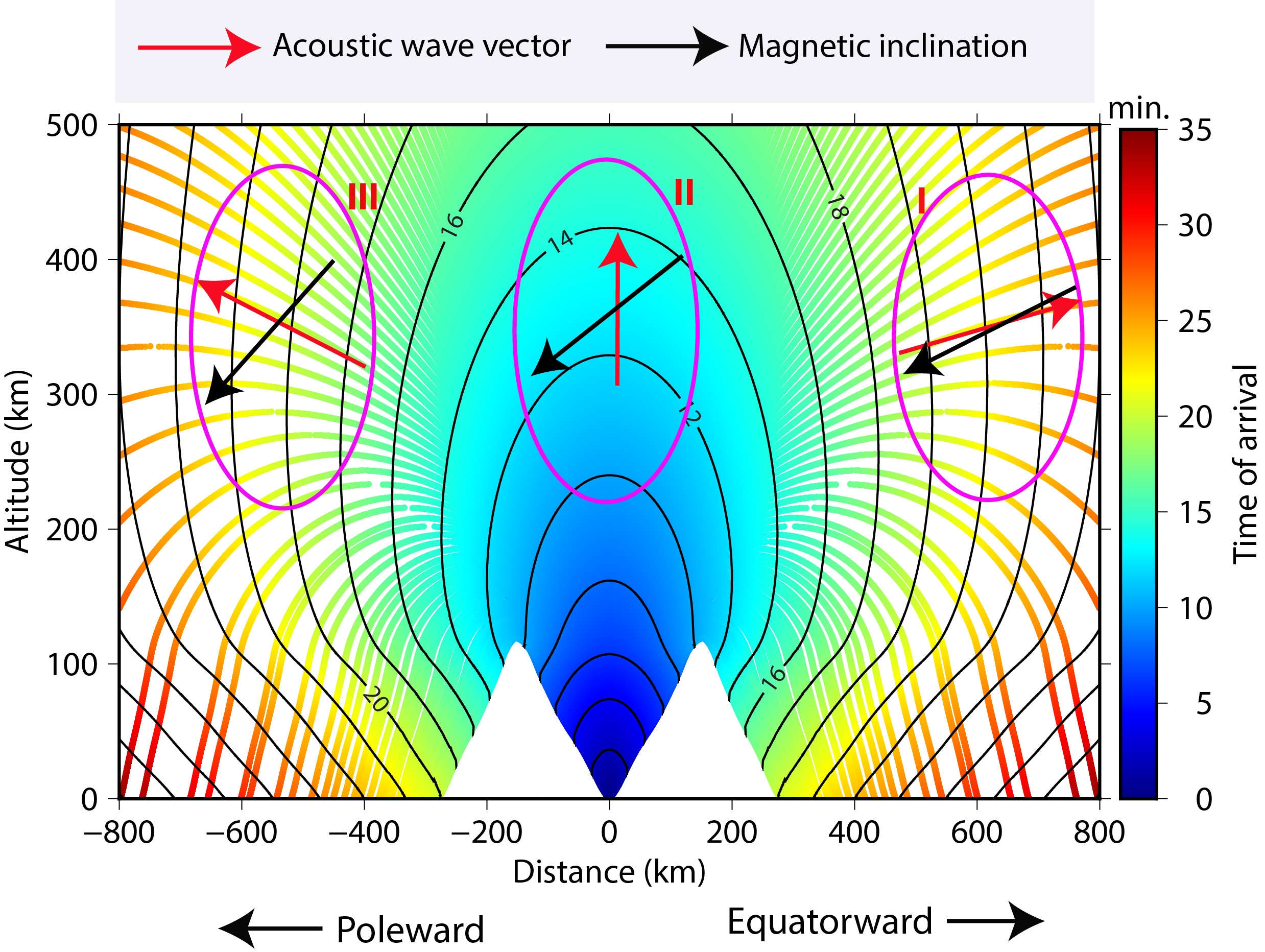


**Figure S3:** 2D schematic showing various orientations between acoustic wave vectors and magnetic inclination around the Trial earthquake source (25^o^N 84^o^E). The rays are modeled at every 0.1° for all possible launch angles (till ~58^o^ from the zenith). Black contour lines demonstrate the wavefronts in terms of arrival time of respective rays at specific altitudes. Red arrows represent the respective acoustic wave vector orientation. Black arrows show the actual magnetic inclination at respective locations. The figure is prepared using the GMT 5.4.4^43^.


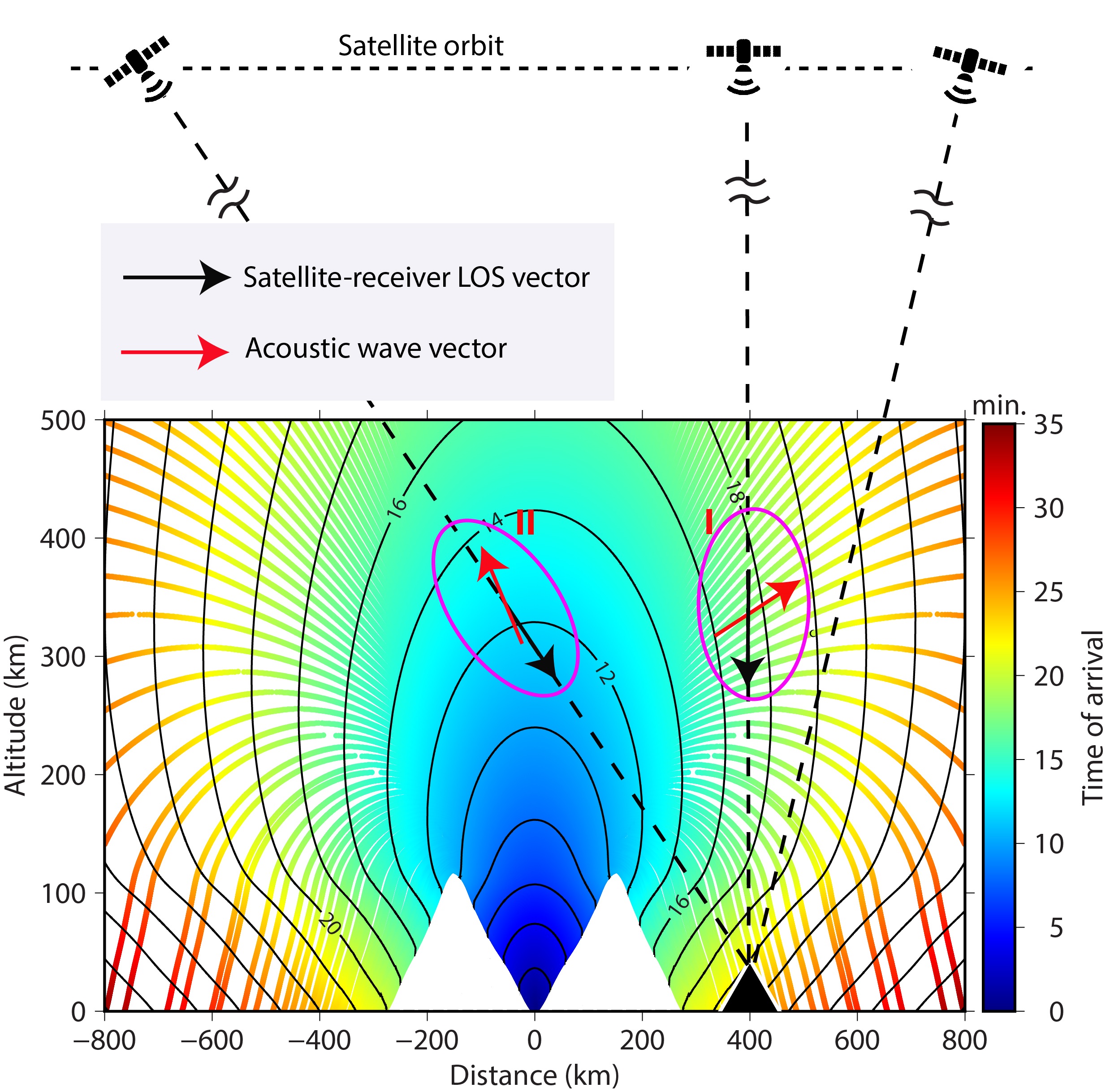


**Figure S4:** 2D realistic schematic showing various orientations between acoustic wave vectors and satellite ling of sight (LOS) from GPS station assumed at 400 km (black triangle) from the Trial earthquake source. The acoustic rays are computed for all launch angles (till ~58^o^ from the zenith) with 0.1° resolution. Black contour lines demonstrate the wavefronts in terms of arrival time of respective rays at specific altitudes. Red arrows represent the acoustic wave vector orientation. Black arrows show the LOSs of three GPS satellites from the assumed station. From case-I, LOS and wave vector are more or less perpendicular which represents the favorable satellite observation geometry. Whilst case-II describes a condition in which the satellite-receiver LOS vector (black arrow) and seismo-acoustic wave vector (red arrow) are oriented at ~180^o^. In such a case, the alternate phases of seismo-acoustic waves integrate at the receiving end and thus are treated as unfavorable satellite observation geometry. The figure is prepared using the GMT 5.4.4^43^.


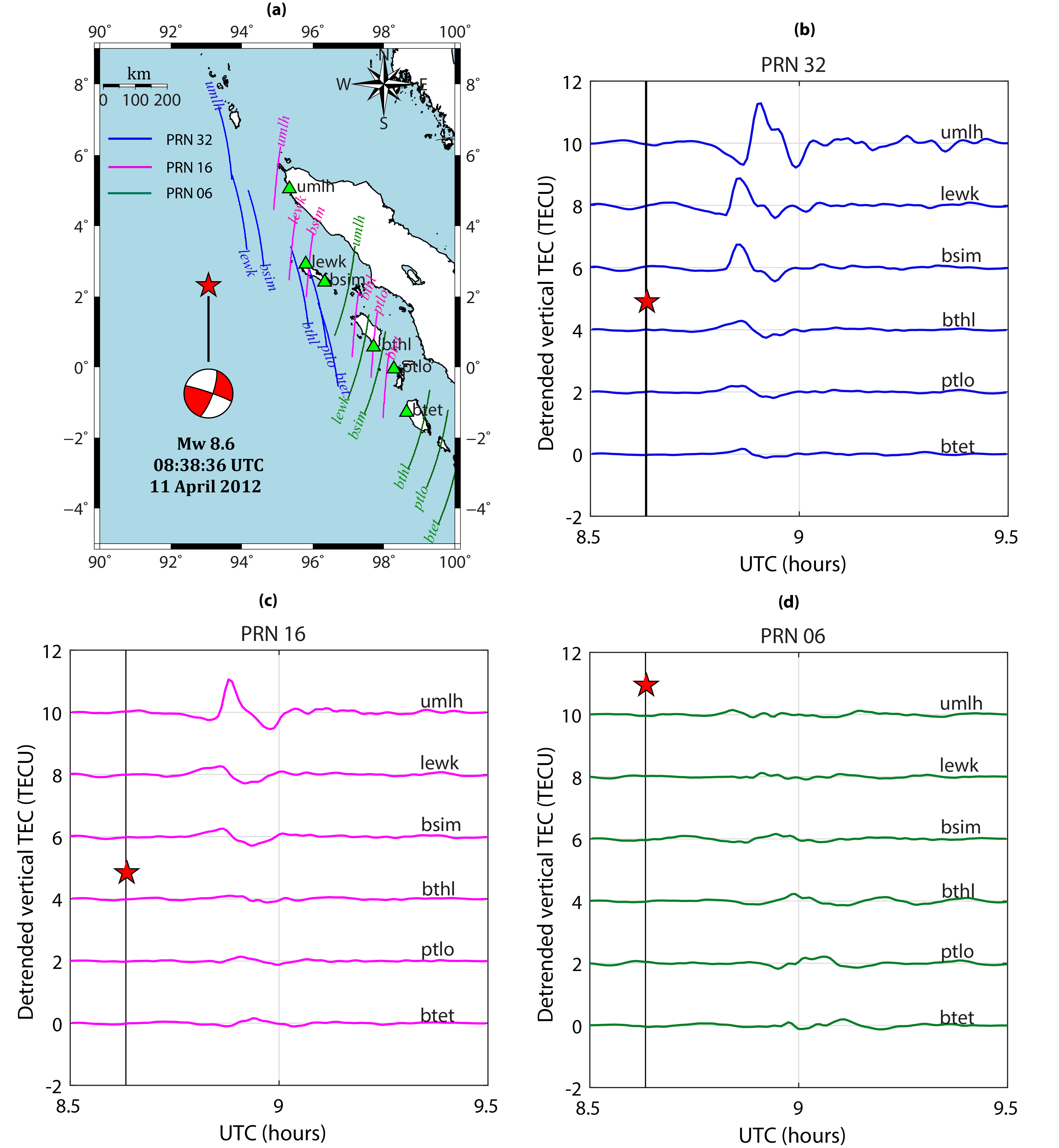


**Figure S5:** (a) Map showing the epicenter location and fault mechanism during the Mw 8.6 11 April 2012 Sumatra (SU) earthquake. IPP tracks of PRNs 32, 16, and 06 at peak density altitude of 350 km during the earthquake occurrence time. The peak density altitude of 350 km is derived based on the IRI-2016 model. The tracks are labeled with their respective observing station names. The locations of stations are shown with triangles. The figure is prepared using the GMT 5.4.4^43^. (b-d) Temporal evolution of CIP as observed by PRNs 32, 16, and 06 respectively. Each time series is labeled with respective recording station names. The red star in each panel shows the location of the epicenter for easy understanding of CIP evolution north and south of the epicenter. Vertical lines in (b-d) show the earthquake onset time.





**Figure S6:** 2D manifestation of NTFM model in terms of GNF, EDF, SGF, and NTFM factor at IPP altitude of 350 km during the SU earthquake. The SGF is estimated for GPS stations of *bsim, btet* and *umlh*. These stations are located at epicentral distance of ~363 km, ~737 km and ~396 km respectively. The figure is prepared using the GMT 5.4.4^43^.


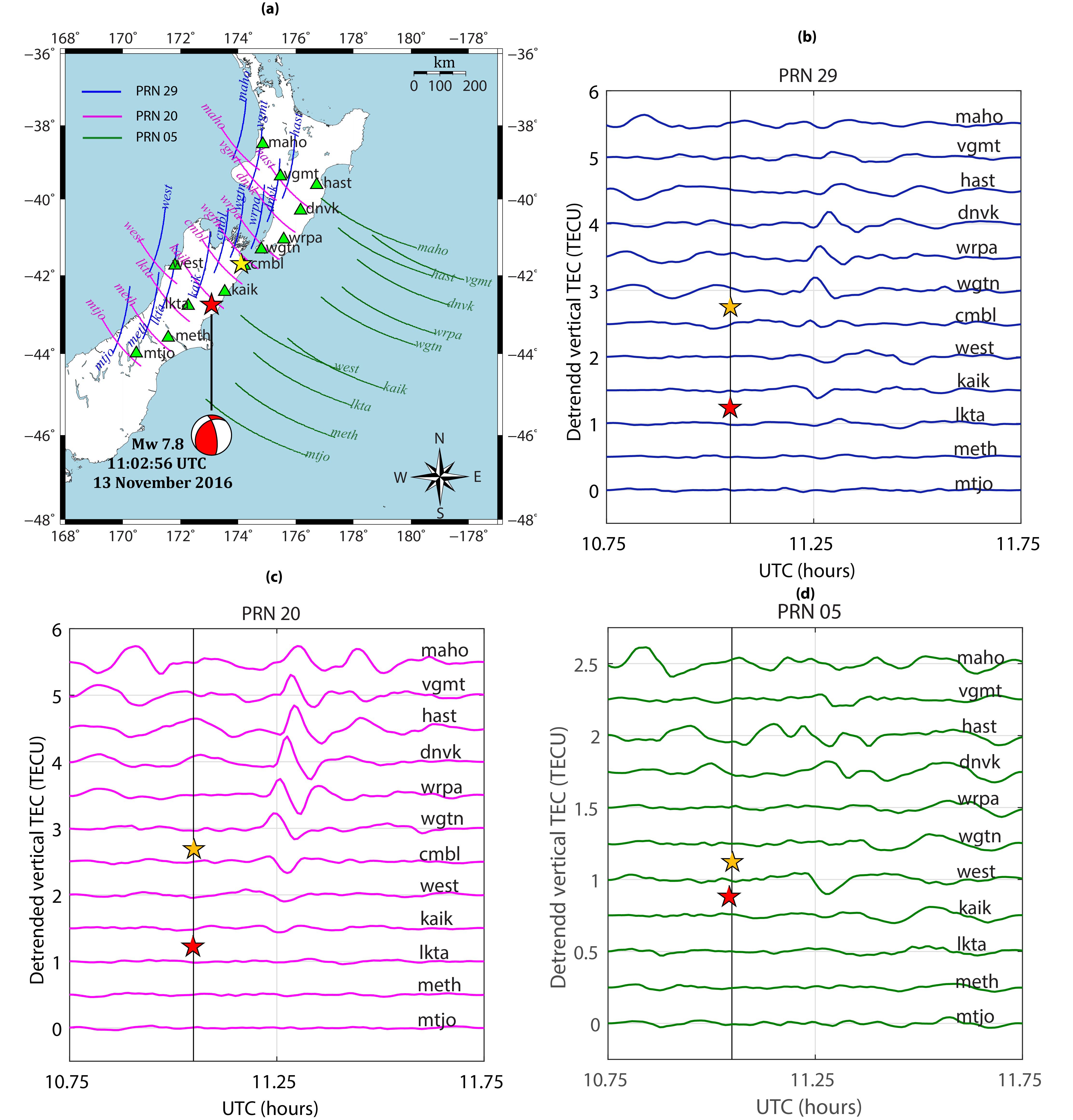


**Figure S7:** (a) Map showing the epicenter location (red star) and the fault mechanism during the Mw 7.8 13 November 2016 New Zealand (NZ) earthquake. The yellow star represents the location of the Campbell Coseismic Thrust Zone (CCTZ)^7^. Bagiya et al., [2018]^7^ demonstrated that ionospheric perturbations during the NZ earthquake were linked to the two distinct surface thrust zones along the rupture direction rather than merely to the coseismic displacements oriented along the rupture. We consider the CCTZ seismic source for the present study. IPP tracks of PRNs 29, 20, and 05 at peak density altitude of 350 km during the earthquake occurrence period. Other information is the same as that of figure S5. The figure is prepared using the GMT 5.4.4^43^. (b-d) Temporal evolution of CIP as observed respectively by PRNs 29, 20, and 05. Yellow star in each time series represents the location of the CCTZ. Other information is same as that of figure S5.





**Figure S8:** 2D manifestation of NTFM model in terms of GNF, EDF, SGF, and NTFM factor at IPP altitude of 350 km during the NZ earthquake. Other information is the same as that of figure S7. The SGF is estimated for GPS stations of *west, dnvk,* and *kaik*. These stations are located at the epicentral distance of ~200 km, ~230 km, and ~94 km respectively. The figure is prepared using the GMT 5.4.4^43^.
